# Bee Pollen Polysaccharide From *Rosa rugosa* Thunb. (Rosaceae) Promotes Pancreatic β-Cell Proliferation and Insulin Secretion

**DOI:** 10.3389/fphar.2021.688073

**Published:** 2021-06-28

**Authors:** Siwen Yang, Yunhe Qu, Jiyu Chen, Si Chen, Lin Sun, Yifa Zhou, Yuying Fan

**Affiliations:** Engineering Research Center of Glycoconjugates Ministry of Education, Jilin Provincial Key Laboratory of Chemistry and Biology of Changbai Mountain Natural Drugs, School of Life Sciences, Northeast Normal University, Changchun, China

**Keywords:** bee pollen polysaccharides, insulin secretion, β-cell proliferation, T1DM, antidiabetic activity

## Abstract

Insufficient pancreatic β-cell or insulin-producing β-cell are implicated in all types of diabetes mellitus. Our previous studies showed bee pollen polysaccharide RBPP-P improves insulin resistance in type 2 diabetic mice by inhibiting liver fat deposition. However, its potential of regulating β-cell function and integrity is not fully known. Herein, we observed that β-cell proliferation (*n* = 10), insulin synthesis (*n* = 5, *p =* 0.01684) and insulin incretion (*n* = 5, *p =* 0.02115) were intensely activated in MIN6 cells when treatment with RBPP-P. In alloxan-induced diabetic mice, oral administration of RBPP-P (*n* = 10) effectively decreased the blood glucose (*p =* 0.0326), drink intake (*p* < 0.001) and urine (*p* < 0.001). It directly stimulated phosphorylation of p38 (*p =* 0.00439), ERK (*p =* 0.02951) and AKT (*p =* 0.0072) to maintain the islet function and mass. Thus, our data suggest that RBPP-P is a natural compound to regulate β-cell proliferation and function, indicating it might have therapeutic potential against type 1 diabetes.

## Introduction

Type 1 diabetes (T1DM) is an autoimmune disorder characterized by the destruction of insulin-producing pancreatic β-cell and resultant hyperglycemia (Wilcox et al., 2016; Katsarou et al., 2017). The treatment of diabetes is mainly based on administration of insulin, the replacement or regeneration of insulin-producing cells, pancreas transplantation (Atkinson et al., 2014). Near perfect control of blood glucose levels can be restored by pancreas or islet transplantation, without the risk of serious hypoglycemic episodes that are associated with intensive insulin therapy. However, current protocols are inadequate to prevent islet rejection long-term, caused harmful side effects and some of them are expensive (Hirshberg et al., 2003). Around 80% of the world’s population uses natural plants and their bioactive compositions for effective, less expensive and low-toxicity treatment (World Health Organization, 2017). These compounds have relative ability to reduce blood glucose while maintaining their growth and secreting properties targeting the endogenous β-cell. Thus, increasing the number of pancreatic β-cell or enhancing the function of islets is an effective way to treat T1DM (Mustafa et al., 2012; Vetere et al., 2014; Alejandro et al., 2015).

The proliferation of β-cell is exquisitely regulated to meet metabolic demand through complex mechanisms that involve the integration and interaction of multiple factors (Bernal-Mizrachi et al., 2014), but the mechanisms are still unclear. Insulin is synthesized by pancreatic β-cell and plays a predominant role in glucose homeostasis. Insulin or insulin-like growth factor (IGF) knockout non-diabetic mice exhibited the decrease of β-cell mass, suggesting that their important roles in regulation of β-cell proliferation (Sabir et al., 2018). Mice have two genes encoding insulin, Ins1 and Ins2. The absence of Ins1 hinders the progress of T1DM, but the absence of Ins2 accelerates the development of T1DM (Babaya et al., 2006). Besides that, the transcription of the insulin gene is regulated by many factors, including MafA, Pdx1 and Neuro D1 (Zhang et al., 2020). Several signaling pathways also participated in β-cell proliferation. Recent studies have suggested crosstalk between AKT and islet-enriched transcription factors (Humphrey et al., 2010; Weng et al., 2020). MAPKs, including ERKs, JNKs and p38-MAPKs, have been found to be associated with cell survival, proliferation and stress response (Trempolec et al., 2013).

Bee pollen is the most popular natural health food in the world, and polysaccharide is the main bioactive components. Studies have shown that bee pollen polysaccharides have different functions, such as immunomodulation (Li et al., 2009), hypoglycemic (Gong et al., 2017), hypolipidemic (Denisow et al., 2016), anti-aging (Graikou et al., 2008) and anti-tumor (Wang et al., 2013). In our previous work, bee pollen polysaccharide from *Rosa rugosa* Thunb. (Rosaceae) were isolated and fractionated into three fractions (RBPP, RBPP-N, RBPP-P), RBPP-P could reduce the levels of blood glucose and lipid in type 2 diabetes (T2DM) mice by alleviating liver steatosis and insulin resistance (Gong et al., 2017). However, the mechanism of RBPP-P in the treatment of protecting the pancreas functions and promoting insulin secretion in T1DM is not fully elucidated. Thus, in this study we evaluated the effects of RBPP-P on β-cell proliferation and pancreas function *in vitro* and *in vivo*.

## Materials and Methods

### Bee Pollen Polysaccharides Preparation and Fractionation

Bee pollen obtained from *Rosa rugosa* Thunb. (Rosaceae) was isolated and fractionated based on our previous report (Gong et al., 2017). Briefly, bee pollen powder was extracted with hot water and polysaccharides (RBPP) were precipitated by ethanol. RBPP was further fractionated by DEAE-cellulose chromatography into the acidic fraction (RBPP-P).

### Bee Pollen Polysaccharides Characterization

The total carbohydrate content was determined using the phenol sulfuric acid method with glucose as the standard. The sugar compositions were analyzed by HPLC, and the structural feature of RBPP-P was analyzed by FT-IR and ^13^C-NMR as described in previous publications (Gong et al., 2017).

### Cell Culture

MIN6 cells (mice pancreatic β-cell line) were obtained from the American Type Culture Collection. MIN6 cells were maintained in DMEM supplemented with 15% fetal calf serum, 2 mM L-glutamine (Gibco, 35050-061), 1 mM pyruvate (Aladdin, S104174) and 285 μM β-mercaptoethanol (Gen-view, GM195) at 37°C in a 5% CO_2_ incubator.

### Cell Viability Assay

For MTT assay, MIN6 cells were seeded at the density of 1.5 × 10^4^ cells on 96-well plate for 24 h. They were grown in pretreatment medium without (as control) or with 0.01, 0.1, and 1.0 mg/ml of polysaccharides for 24 h. The media were removed and 100 μl MTT (0.5 mg/ml) was added and incubated for additional 4 h. For dissolving formazon, 50 μl of 20% SDS/0.04% HCl solution was added to each well, and incubated in 37°C overnight. The absorbance at 570 nm was measured using a microplate reader (Biotek, United States). Cell proliferation under all the conditions was expressed as a percentage of the control, which was set at 100%.

### Ki-67 Immunostaining

MIN6 cells were seeded on 24-well tissue culture plate at 1.5 × 10^5^ cells and incubated for 24 h. The cells were grown for 24 h in pretreatment medium without (as control) or with 0.1 mg/ml of polysaccharides. After that, cells were gently washed with PBS and then fixed for 20 min at room temperature in 4% paraformaldehyde. Fixed cells were permeabilized in 0.1% Triton X-100/PBS for 15 min and then blocked with 2% BSA/5% bovine serum in PBS at room temperature for 20 min. Afterward, cells were incubated with a rabbit monoclonal antibody against Ki-67 (CST, 9129S, 1:500) for 1 h. After washing with PBS, cells were incubated with Alexa Flur™ 594-conjugated goat anti rabbit antibody (Invitrogen, A11072, 1:100) and DAPI (1:500) for 40 min. The images were taken using epi-fluorescence microscope (Olympus BX51) with ×40objective.

### Insulin Secretion Assay

MIN6 cells were seeded in 48-well plate at 5 × 10^4^ cells for 24 h, followed by 0.1 mg/ml polysaccharides or 30 mM KCl for 24 h. Then cells were incubated in KRB balanced buffer (115 mM NaCl, 4.8 mM KCl, 2.5 mM CaCl_2_, 1.2 mM MgSO_4_, 1.2 mM KH_2_PO_4_, 20 mM NaHCO_3_, and 16 mM HEPES; pH 7.4) containing 0.2% BSA for 2 h. Medium was then replaced with KRB containing 5.5 mM glucose, 5.5 mM glucose plus 30 mM KCl or 5.5 mM glucose plus 1.0 mg/ml of different polysaccharides for 1 h, respectively. Supernatant was collected and insulin content was measured by ELISA (Innovation Beyond Limits, Germany). The cells were lyzed with lysis buffer (Boster, AR0102) for measurement of total protein content. The insulin secretion was defined as insulin content/protein content.

### qRT-PCR Assay

qRT-PCR was established as described previously (Yang et al., 2020). MIN6 cells were isolated using TRIZOL (Invitrogen) and cDNA was generated from 1 μg of RNA using M-MLV reverse transcriptase (Promega, Fitchburg, WI, United States). The 20 μl of PCR mixture contained 1 μl of cDNA. Real-time PCR assays were conducted with a LC480 Light Cycler (Roche, Germany) using the applied primer sequences listed Table 1. PCR amplification was conducted as following: denaturation at 95°C for 30 s, annealing at 56°C for 30 s, and extension at 72°C for 45 s. GAPDH were used as internal standards for mRNAs. Relative expression of genes was determined using a comparative method (2^−△CT^).

**TABLE 1:** Primers used in the quantitative real-time PCR experiment.

Gene name	Forward (5′-3′)	Reverse (5′-3′)
*Insulin1*	CAC​TTC​CTA​CCC​CTG​CTG​G	ACC​ACA​AAG​ATG​CTG​TTT​GAC​A
*Insulin2*	GCT​TCT​TCT​ACA​CAC​CCA​TGT​C	AGC​ACT​GAT​CTA​CAA​TGC​CAC
*MafA*	AGG​AGG​AGG​TCA​TCC​GAC​TG	CTT​CTC​GCT​CTC​CAG​AAT​GTG
*Pdx1*	CCC​CAG​TTT​ACA​AGC​TCG​CT	CTC​GGT​TCC​ATT​CGG​GAA​AGG
*Gapdh*	AGG​TCG​GTG​TGA​ACG​GAT​TTG	TGT​AGA​CCA​TGT​AGT​TGA​GGT​CA

### Western Blot

Pancreas were lyzed in lysis buffer, containing 50 mM Tris-Cl (pH 7.5), 150 mM NaCl, 1 mM EGTA, 1% TritonX-100, 100 mM NaF, 10 mM Na_4_P_2_O_7_ and 1 mM PMSF. The supernatants were collected after centrifugation, and the protein concentration was determined using the Coomassie Brilliant Blue (BBi, A610037-0025) assay. 30 μg tissue lysate was detected by western blot and probed for antibodies against phospho-p38 (Thr180/Tyr182) antibody (CST, 9215), p38 antibody (CST, 9212), phospho-ERK1/2 (Thr202/Tyr204) antibody (CST, 9101), ERK1/2 antibody (CST, 9107), phospho-Akt (Ser473) antibody (CST, 9271), Akt antibody (CST, 9272), actin (BD Biosciences, 612,657). Quantification was performed using the ImageJ software (Rockville, MD, United States).

### Animal Treatment

Male C57BL/6J mice were obtained from GemPharmatech Co., Ltd. (Nanjing, China). All experiments were approved by the Animal Care and Use Committee of Northeast Normal University (SYXK 2018-0015). The mice were housed in a temperature-controlled facility (21°C, 12 h light/12 h dark cycle, 60–70% humidity), provided standard laboratory chow and water, and were subjected to treatment at 5 weeks of age.

### Induction T1DM

To induce T1DM, the animals in the experimental group were fasted for 24 h, able to freely asscess water. Mice were injected intraperitoneally with fresh prepared alloxan (ALX, 150 mg/kg; sigma, A7413) saline solution for consecutive five days. Fasting blood glucose of the animals were measured and only the mice with blood glucose levels above 200 mg/dl were used for the experiments.

### Experimental Design

Thirty mice were randomly divided into three groups (*n* = 10) and treated oral daily for 28 days. Group I: normal control; Group II: ALX control; Group III: ALX mice treated with RBPP-P (100 mg/kg). Fasting blood glucose (FBG), body weight and food intake were measured each week of the experiment. Urine volume and water consumption of animals were evaluated on the 28th days of the experiment. At the end of study, blood and organ samples were collected for the determination of biochemical parameters.

### Serum Biochemical Analyses

Mice were analysis for insulin after overnight fasting when the animal assays finished. Serum were abtained and insulin level was measured using competitive enzyme-linked immunosorbent assay kits (Innovation Beyond Limits, Germany) according to the manufacturer’s instructions. Ketone bodies was measured using competitive enzyme-linked immunosorbent assay kits (Zhenke biology, China) according to the manufacturer’s instructions.

### Immunohistochemistry

Based on our previous report (Fan et al., 2017), the pancreas was fixed in 4% paraformaldehyde and then a small part of the complete pancreas was cut. The pancreas tissues were soaked in 15% sucrose and 30% sucrose dehydrated overnight, respectively. The dehydrated tissues were embedded in OCT Compound (SAKURA, 4583) and frozen in −80°C. The tissues were subsequently sliced into 8 μm sections using freezing microtome (LEICA CM1850UN).

Sections were fixed with 4% paraformaldehyde for 20 min. Then permeabilized with 0.5% Triton X-100/SDS/PBS for 20 min, and blocked with 5%FBS/PBS/1% BSA for 3 h at room temperature. The slides were then stained with insulin (CST, 3014) and glucagon (Boster Biological Technology, BM1621) antibody overnight at 4°C. After washed, slides were dark incubated with FITC-conjugated goat anti-rabbit IgG (ABclonal, AS011) and Cy3-conjugated goat anti-rabbit IgG (ABclonal, AS008) for 2 h at room temperature. Sections were then washed with PBST and PBS for 5 min, followed incubated with Hoechest 33342 for 10 min at room temperature. After three washes with PBS, the slides were covered with fluorescence decay resistant medium (Boster, AR1109). Pancreatic sections were imaged using a light microscopy (Olympus BX51). The insulin-positive area and pancreatic area were measured using ImageJ software (Rockville, MD, United States).

### Statistical Analysis

The results were expressed as the means ± s.d. Statistical analysis of the data was performed using Student’s *t*-test and two-way repeated-measures ANOVA with Dunnett’s post-hoc test (IBM SPSS Statistics 17.0, Armonk, NY, United States). All experiments were repeated at least three times. The level of significance was set at *p < 0.05, **p < 0.01, ***p < 0.001.

## Results

### Preparation and Characterization of RBPP-P

**FIGURE 1:**
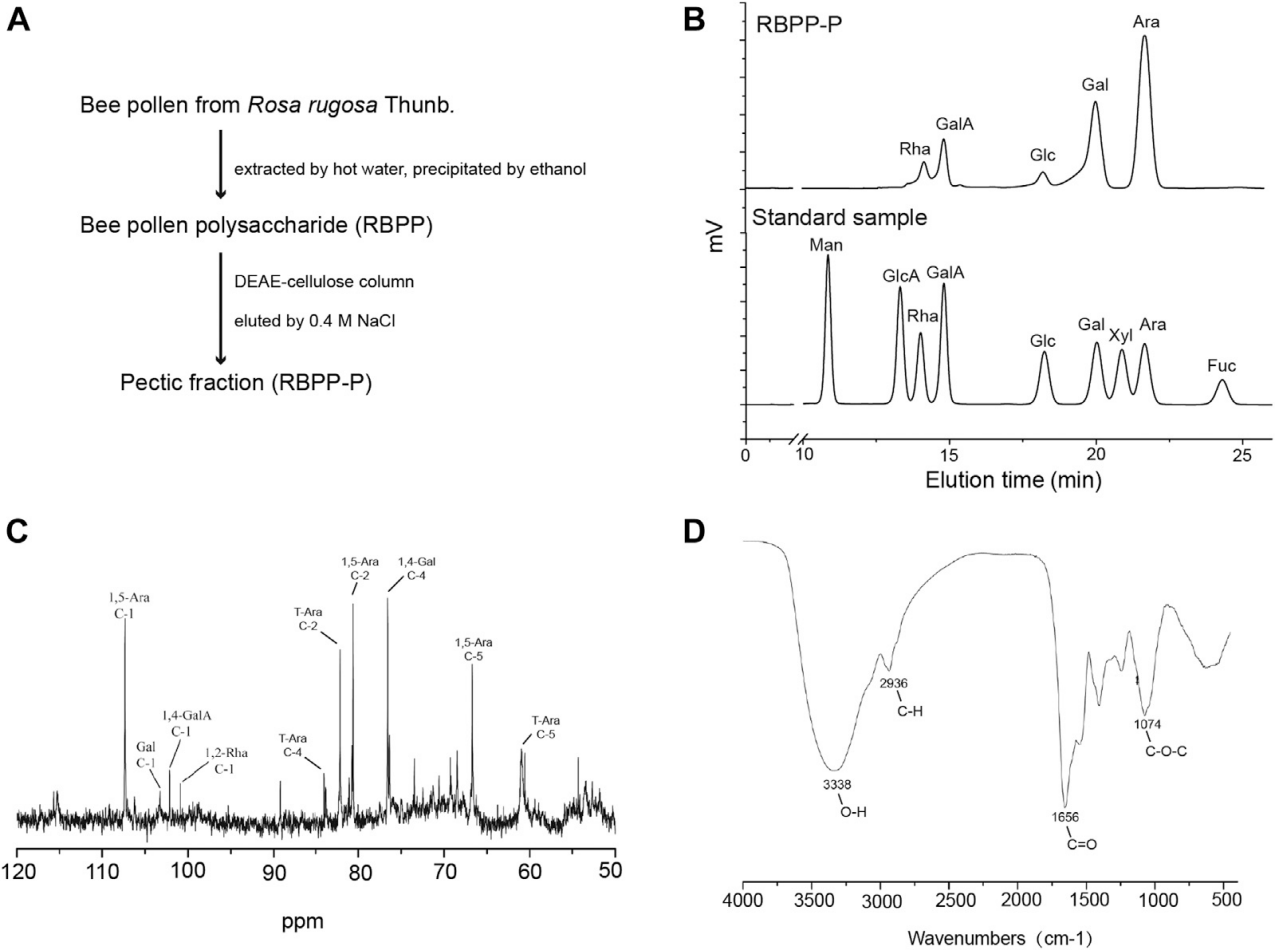
Preparation and characterization of bee pollen polysaccharide. **(A)** The procedure used to prepare RBPP-P from *Rosa rugosa* Thunb. (Rosaceae). **(B)** Analysis of the monosaccharide composition of RBPP-P using high performance liquid chromatography (HPLC). **(C)** Analysis of chemical structure of RBPP-P using ^13^C-nuclear magnetic resonance (^13^C-NMR). **(D)** Analysis of the chemical structure of RBPP-P using fourier transform-infrared spectroscopy (FT-IR).

Bee pollen polysaccharide (RBPP) was obtained from *Rosa rugosa* Thunb. (Rosaceae) by hot water extraction and ethanol precipitation. It was then separated by DEAE-Cellulose column and eluted with 0.4 M NaCl into an acidic fraction (RBPP-P), contains 93.6% of total carbohydrate (Figure 1A). Monosaccharide composition analysis by HPLC showed that RBPP-P is mainly composed of Ara (arabinose, 50.6%), Gal (galactose, 22.6%), GalA (galacturonic acid, 12.8%) and Rha (rhamnose, 5.6%), Glc (glucose, 4.4%), indicating that the polysaccharides we extracted are consistent with before (Figure 1B). Structural features of RBPP-P were characterized by analyzing ^13^C-NMR and FT-IR spectrum (Figures 1C,D), indicated that RBPP-P contained more proportion of arabinogalactan (AG) fragments and small amounts of rhamnogalacturonan I (RG-I) and homogalacturonan (HG) domains.

### RBPP-P Activated β-Cell Proliferation and Insulin Synthesis *in vitro*

**FIGURE 2:**
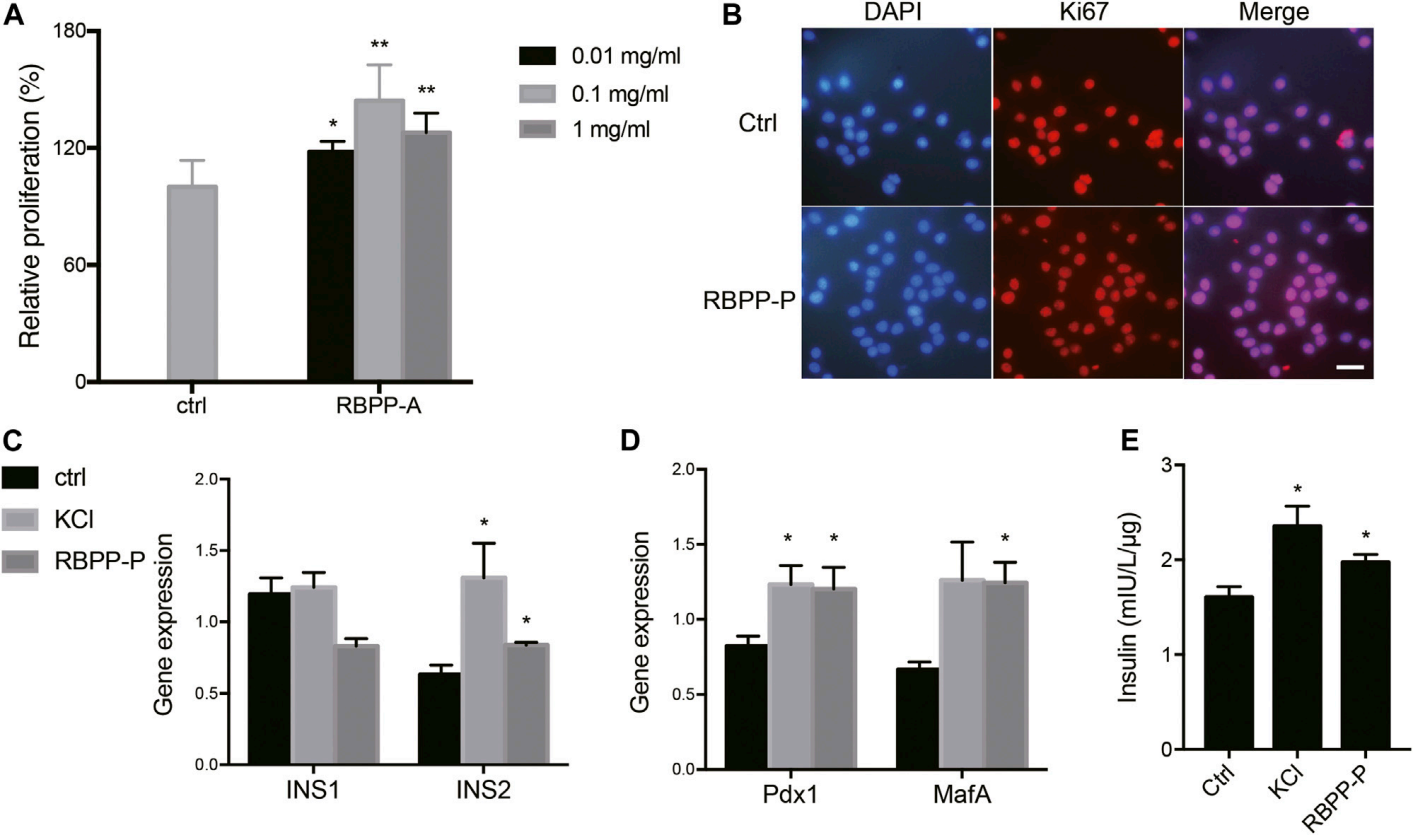
Effects of RBPP-P on cell viability, insulin gene expression and insulin secretion. **(A)** RBPP-P promoted cell proliferation by MTT assay (*n* = 5). **(B)** RBPP-P promoted cell proliferation by Ki-67 immunostaining (*n* = 10). Scale bar, 100 μm. **(C)** qRT-PCR was used to evaluate the Ins1 and Ins2 levels in Min6 cells (*n* = 5). **(D)** qRT-PCR was used to evaluate the Pdx1 and MafA levels in Min6 cells (*n* = 5). mRNA levels were normalized to Gapdh mRNA. **(E)** Insulin secretion levels in Min6 cells (*n* = 5). All data are expressed as mean ± s. d. *p < 0.05, **p < 0.01, vs. control.

To investigate the polysaccharide caused proliferative effect, MIN6 cells were treated with increasing concentration of bee pollen polysaccharides for 24 h. Cell viabilities were assessed by MTT assay. Concentration-effect relationship indicated that the enhancement of cell proliferation by RBPP-P was 20.8, 45 and 30.3% at the concentration of 0.01, 0.1 and 1.0 mg/ml (Figure 2A). Followed, carried out Ki-67 immunostaining which cells stained with Ki-67 (in red) and DAPI (in blue) under epi-fluorescence microscope. Ki-67 only stained the proliferating cells in the active cell cycle, but not the resting cells. When compared with control cells, RBPP-P appeared more cells in active cell cycle (Figure 2B). The results were consistent with that of MTT assay that RBPP-P might play a role on stimulation cell proliferation.

We further investigated the potential role of RBPP-P in regulation of insulin expression. When compared with control cells, 0.1 mg/ml of RBPP-P activated the Ins2 mRNA in Min6 cells, but not Ins1 (Figure 2C). Because MafA is a master regulator of the insulin gene, we determined the effect of RBPP-P on its expression and found that MafA was strongly increased in potassium chloride (KCl) and RBPP-P cells (Figure 2D). Pdx1 (pancreas-duodenum homeobox1) is a key transcription factor which activated insulin synthesis and stimulated pancreatic beta cell proliferation. As expected, the expression of Pdx1 was stimulated with RBPP-P treatment (Figure 2D).

Next, we performed glucose stimulated insulin secretion. KCl treated at 30 mM induced strong insulin secretion in cells. Compared to control cells, RBPP-P also exhibited insulin release at basal glucose. (Figure 2E). Collectively, these data supported that RBPP-P modulates MafA and Pdx1 protein and activates Ins2 gene expression, showed its ability to activate β-cell proliferation and insulin synthesis.

### RBPP-P Reduced Blood Glucose and Alleviated the Symptoms of Diabetes in ALX-Induced Diabetic Mice

**FIGURE 3:**
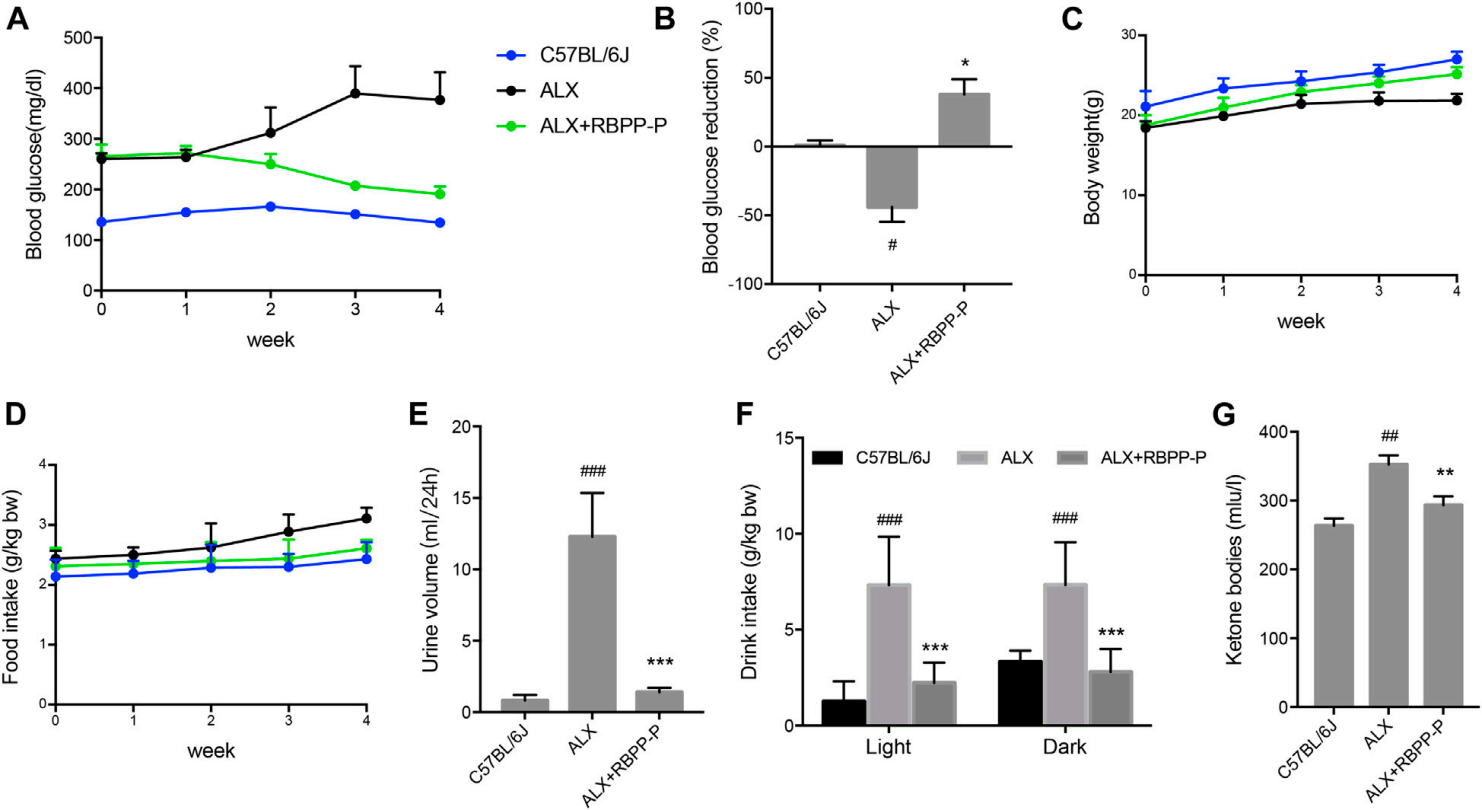
Treatment with RBPP-P for 4 weeks improved hyperglycaemia and daily indicates in ALX mice (*n* = 10). Fasting blood glucose **(A)** and blood glucose reduction **(B)** were measured. **(C)** Body weight **(D)** Food intake. **(E)** Urine volume **(F)** Water intake **(G)** Serum ketone bodies. All data are expressed as mean ± s. d. ^*#*^ *p* < 0.05, ^*##*^ *p* < 0.01, vs. C57BL/6J mice; *p < 0.05, **p < 0.01, vs. ALX mice.

We additionally investigated whether RBPP-P activation in β-cells reverses the hyperglycemia induced by alloxan (ALX). Intraperitoneal injection of alloxan monohydrate (150 mg/kg) caused over 2-fold elevation of blood glucose level, which was maintained over a period of 4 weeks. Daily oral treatment with 100 mg/kg of RBPP-P for 4 weeks, led to a significant decrease in fasting blood glucose (FBG) levels (Figures 3A,B). The results were in accordance with the previous results that RBPP-P had an anti-diabetes potential in HFD-mice.

Weight loss, polyphagia, polyuria and polydipsia are the main features of T1DM. Although administration of RBPP-P had the potential to block the ALX effect on body weight (Figure 3C), RBPP-P failed to reverse the increase in food intake after ALX damaged (Figure 3D). However, after treatment for 4 weeks, RBPP-P mice showed significantly decrease in water consumption (Figure 3E), urinary volume (Figure 3F) and ketone bodies level (Figure 3G) compared with ALX mice. This suggested that RBPP-P has shown a significant antidiabetic effect in alloxan-induced diabetic mice.

### RBPP-P Restored Islet Function of ALX-Mice

**FIGURE 4:**
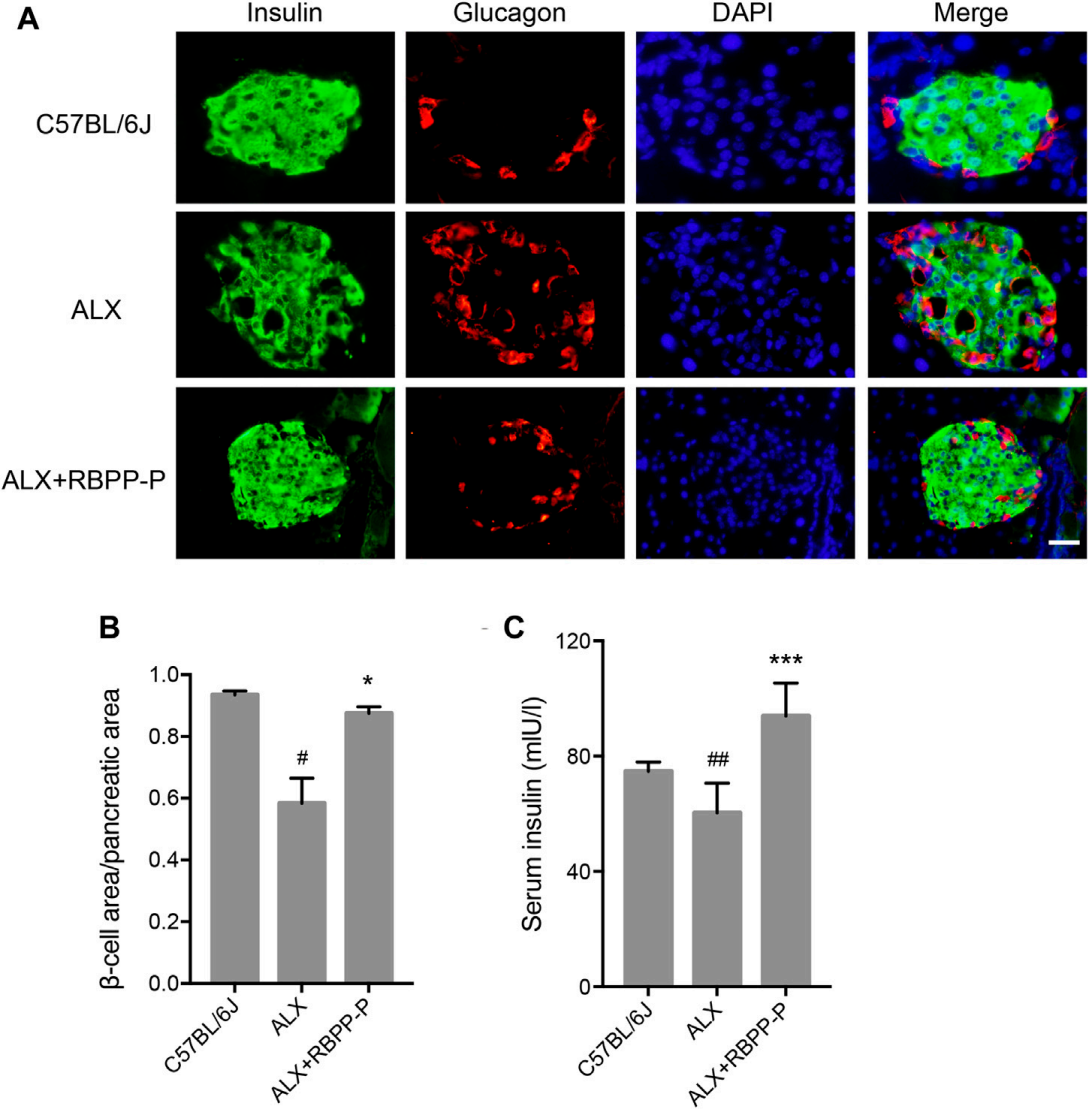
Effects of the RBPP-P on islet histology. **(A)** Immunofluorescence (IF) staining for insulin (green) and glucagon (red). Scale bar, 100 μm. **(B)** β-cell area/pancreatic area ratio, measured by immunofluorescence staining (*n* = 5). **(C)** Insulin levels in serum (*n* = 10). All data are expressed as mean ± s. d. ^*#*^ *p* < 0.05, vs. C57BL/6J mice; *p < 0.05, vs. ALX mice.

Islet β-cell dysfunction leads to insulin insufficiency, a contributor to the onset of hyperglycemia in T1DM. We performed immunofluorescent staining of pancreatic sections using specific antibodies against insulin and glucagon. The C57BL/6J group showed islets of normal size, however, the ALX group observed reduction in the size of the insulin-expressing β-cells, enlarged the area of glucagon-expressing α-cells, and the disappearance of islet borders. This damage was considerably reduced after the administration of the RBPP-P, which was highly expressed of β-cells, and decreased α-cells located in the outer rim of the pancreatic islets (Figure 4A). It was demonstrated that RBPP-P displayed significantly increased the β-cell area/pancreatic area ratio with ALX group (Figure 4B). In addition, the insulin level was significantly increased of the RBPP-P treatment compared to the ALX mice (Figure 4C). These results showed RBPP-P preserved structural integrity of pancreatic and restored islet function.

### RBPP-P Stimulates MAPK and AKT Signaling Pathways

**FIGURE 5:**
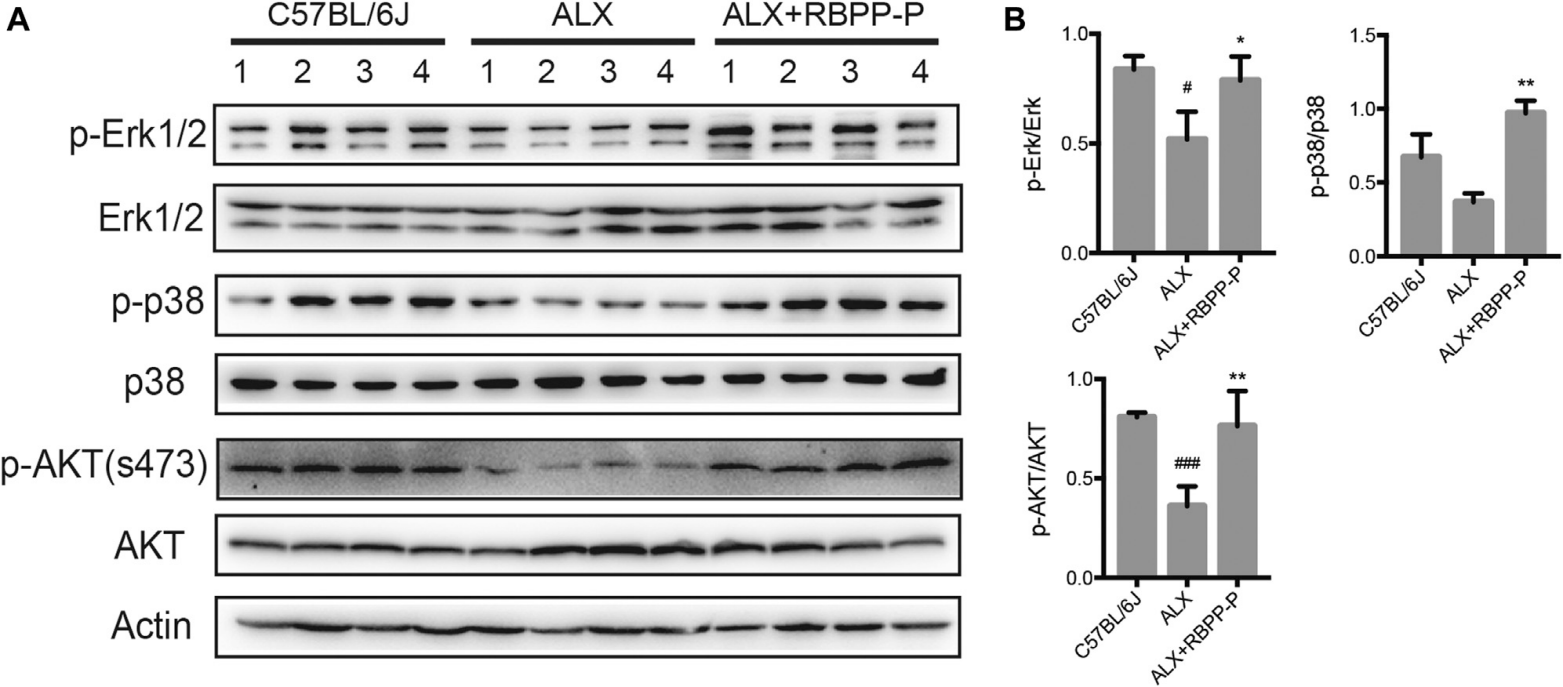
The expression of AKT and MAPK signaling pathways in RBPP-P mice pancreas. **(A)** The MAPK and AKT signaling pathways were analyzed using western blotting **(B)** Band density was measured using the ImageJ. All data are expressed as mean ± s. d. ^*#*^ *p* < 0.05, ^*###*^ *p* < 0.001, vs. C57BL/6J mice; *p < 0.05, **p < 0.01, vs. ALX mice.

MAPK and AKT pathways have been shown to play key roles in regulation of the cell proliferation in mammalian cells. To elucidate if these classic pathways involved in RBPP-P reversed hyperglycemia in ALX-induced mice, we tested the expression levels focusing on pancreatic islet. Results showed that RBPP-P treated mice exhibited increased the phosphorylated levels of p38, ERK1/2 and Akt (S473) compared to C57BL/6J mice (Figures 5A,B). Taken together with other results in mice, suggested that AKT and MAPK signaling induction by RBPP-P plays a critical role in regulation of hypoglycemia.

## Discussion

The relevance between diabetes and β-cell dysfunction is a fundamental research topic for the development of new therapies increasing insulin secretion and optimizing metabolism of T1DM. Here, we prepared the active bee pollen polysaccharide RBPP-P from *Rosa rugose*, attempted to examine the effect of anti-T1DM and possible molecular mechanism of RBPP-P improving β-cell function.

Deregulation of a subset of transcriptional regulators in pancreatic β-cell is underlined a fundamental cause of β-cell dysfunction leading to T1DM (Harmon et al., 2005). The results that RBPP-P activated Ins2 gene expression by modulating MafA1 and Pdx1, which are critical transcription factors for insulin gene expression and secretion. Thus, RBPP-P promotes insulin production and induces expression of transcription regulators maintaining β-cell maturity.

T1DM is a metabolic disease characterized by impaired insulin secretion and resultant hyperglycemia (Lebovita, 2010). Weight loss, polyphagia, polyuria and polydipsia are the main features of T1DM (Bibak et al., 2014). Numerous studies have shown that alloxan destroys insulin-producing β-cells, a chemical commonly used in laboratories to make diabetic models (Katahira et al., 2020). Administration of RBPP-P (100 mg/kg) for 4 weeks showed marked hypoglycemic effect in ALX-induced diabetic mice. RBPP-P significantly reduced the signs of polydipsia and polyuria seen in diabetic mice, which could be the result of better control of hyperglycemia in T1DM. However, body weight and food intake were not significantly different between RBPP-P and ALX animals. Studies have reported that polysaccharides could restore damaged islets (Yang et al., 2019), histological examination in our studies showed RBPP-P significantly reversed the damage in islet induced by alloxan, increased the β-cell area/pancreatic area ratio. These results are consistent with our previous work of RBPP-P carried out on the model of diabetes induced by HFD (Gong et al., 2017).

Cell proliferation, growth and survival are mediated by complex intracellular signaling network including several major pathways: PI3K/Akt and MAPK (p38, Erk and JNK) (Zhang and Liu, 2002; Song et al., 2019; Xie et al., 2021). We have observed that RBPP-P treatment increases the phosphorylation of p38, Erk1/2 and AKT in pancreas. Increasing evidence showed polysaccharides regulated cell proliferation via one of these signaling pathways or even several pathways synchronously. *Lycium barbarum* polysaccharide induced IGF-1-stimulated proliferation of MCF-7 cell through the ERK pathway (Huang et al., 2012). *Capsosiphon fulvescens* polysaccharides regulated effector β-catenin and Erk1/2 activation induced rat small intestinal epithelial cell proliferation (Go et al., 2011). IGF stimulated INS-1 cell proliferation via its downstream MAPK, which could phosphorylate ERK1/2 and p38 (Trumper et al., 2001). Thus, we speculated RBPP-P could stimulate growth factor to indirectly act on pancreatic beta cell, perhaps it may active multiple growth factors synchronously.

Based on these findings, we have explained how RBPP-P modulates β-cell production and β-cell function. RBPP-P activated key transcription factor MafA and Pdx1 in β-cells, increasing insulin expression and insulin secretion. RBPP-P also upregulates the phosphorylation levels of p38, ERK and AKT, promotes β-cell proliferation. Oral administration of RBPP-P showed potential hypoglycemic activities, improving the symptoms of diabetes, protecting the pancreas in T1DM mice, which maintains β-cell function and glucose homeostasis. Thus, RBPP-P could be used as a potentially natural functional food for the prevention and treatment of T1DM. Further pharmacological and biochemical studies are underway to elucidate the specific mechanism of RBPP-P for β-cell insulin secretion.

## Data Availability

The raw data supporting the conclusion of this article will be made available by the authors, without undue reservation, to any qualified researcher.
